# Phylogenetic analysis of the complete mitochondrial genome of the orange-winged sulphur butterfly *Dercas nina* Mell 1913 (Insecta: Lepidoptera: Pieridae: Coliadinae)

**DOI:** 10.1080/23802359.2024.2427109

**Published:** 2024-11-13

**Authors:** Arlene M. Agcaoili, Nabiha Ameena, Dexter Andres, Rhey Caners, Manishvinder K. Chahal, Nicole J. Croitor, Gabrielle M. David, Kaesy L. Enns, Oleksandra Fedorova, Hannah A. Garber, Sarah D. Gregoire, Tenley E. Ilnisky, Annie Jiang, Anthony Kozak, Feryal Ladha, Alexandria Martin, Mary A. McAuley, Liam R. McEachern, Cassidy McNeill, Senudi D. Nanayakkara, Ngoc Thao Vy Nguyen, Gaeun Park, Deanna K. Peters, Madison N. Poitras, Jolene Potts, Dhruvi V. Prajapati, Camille D. Prefontaine, Ravindu V. Rajapaksha, Pratyaksh Singhal, Cedey Souriyavong, Colby Stoker, Kayla R. Talabis, Yantong Tan, Jasmin L. Tang, Kailey W. Tkach, Ashley J. Tohms, Cameron G. Tramley, Josh Treftlin, Diya Ukani, Ethan A. Vallelly, Patrick V. Wiens, Carissa Yee, Ke Yu, Jeffrey M. Marcus, Living Prairie Mitogenomics Consortium

**Affiliations:** Department of Biological Sciences, University of Manitoba, Winnipeg, Canada

**Keywords:** Illumina sequencing, mitogenomics, genome skimming

## Abstract

*Dercas nina* Mell 1913 (Pieridae) is a little-studied butterfly species endemic to China that flies primarily in the forest canopy. Genome skimming by Illumina sequencing allowed assembly of 146,702 reads for complete 1471.3-fold mean coverage of the circular 15,264 bp mitogenome from *D. nina* consisting of 82.1% AT nucleotides. A gene order typical of butterflies was recovered consisting of 13 protein-coding genes, 22 tRNAs, two rRNAs, and a predicted control region. The *Dercas nina COX1* open reading frame begins with atypical start codon CGA. Six protein-coding genes (*COX1*, *COX2*, *ND2*, *ND3*, *ND4*, *ND5*) with single-nucleotide (T) stop codons, and two protein-coding genes (*ATP6*, *ATP8*) with two-nucleotide (TA) stop codons encoded in the DNA were inferred to be completed by adenine nucleotides from the Poly-A tail of the mRNA. Bayesian’s phylogenetic reconstruction places the *D. nina* and *D. lycorias* mitogenomes as sister clades. *Dercas* mitogenomes were sister to those from genus *Colias* in the monophyletic subfamily Coliadinae. The mitogenome phylogeny is consistent with previous molecular phylogenetic hypotheses based on other markers, but differs somewhat from a morphology-based hypothesis that suggested that *Dercas* was more closely related to genus *Gonepteryx*. This may falsify the hypothesis or may instead reflect mitochondrial-nuclear phylogenetic discordance.

## 1. Introduction

The pierid butterfly genus *Dercas* Doubleday [1847] (Insecta: Lepidoptera: Pieridae: Coliadinae) is currently thought to be comprised of five species found in south and southeast Asia (Schulze and Fiedler 1997). These butterflies fly primarily high in the forest canopy and consequently, many aspects of their biology are not well-studied, but they do visit the ground to take up water and nutrients from damp soils (Schulze and Fiedler 1997; Schulze et al. 2001). *Dercas lycorias* adults are known pollinators of *Hedychium coccineum* (Zingiberaceae) flowers (Gao et al. 2012). *Dercas* larvae feed in the forest canopy on the leaves of the woody vines in genus *Dalbergia* (Fabaceae). This larval feeding pattern has been suggested as a synapomorphy for the genus *Dercas*, separating it from the related and morphologically similar genus, *Gonepteryx* (Schulze and Fiedler 1997; Bozano et al. 2016). *Dercas nina* Mell 1913 is a mostly yellow butterfly found primarily in the middle latitudes of China. The Chinese common name of this species is 橙翅方粉蝶 (Liu 2019), which in English translates to ‘orange-winged pierid butterfly’. In keeping with the Chinese name, we propose ‘orange-winged sulphur butterfly’ as the English common name for *D. nina*. Here, we report the complete mitochondrial genome sequence of *D. nina* assembled from Illumina sequence libraries.

This mitogenome was assembled through a course-based inquiry exercise (Marcus et al. 2010), conducted by the undergraduate students making up the Living Prairie Mitogenomics Consortium, which assembles previously undocumented arthropod mitogenomes for improved DNA-based species identification and phylogenetics (Living Prairie Mitogenomics Consortium 2017, 2018, 2019, 2022; Marcus 2018; Ajibola et al. 2019; Aguila et al. 2021). Student participants assembled and annotated the mitogenome sequence for presentation here. This strategy for sequencing and annotation increases knowledge of mitogenome structure and evolution, while simultaneously training junior scientists in the techniques required for this work.

Worldwide, there are approximately 19,500 species of butterflies (Kawahara et al. 2023), and many species have few genomic resources available. Producing these resources for understudied species and training personnel to manipulate and analyze genomic resources are the main goals of the Living Prairie Mitogenomics Consortium. The *D. nina* mitogenome shows many features typical of butterflies, making it a good exemplar for students learning to assemble and annotate these sequences.

## 2. Materials and methods

### 2.1. Sample collection and preservation

**Figure 1.**
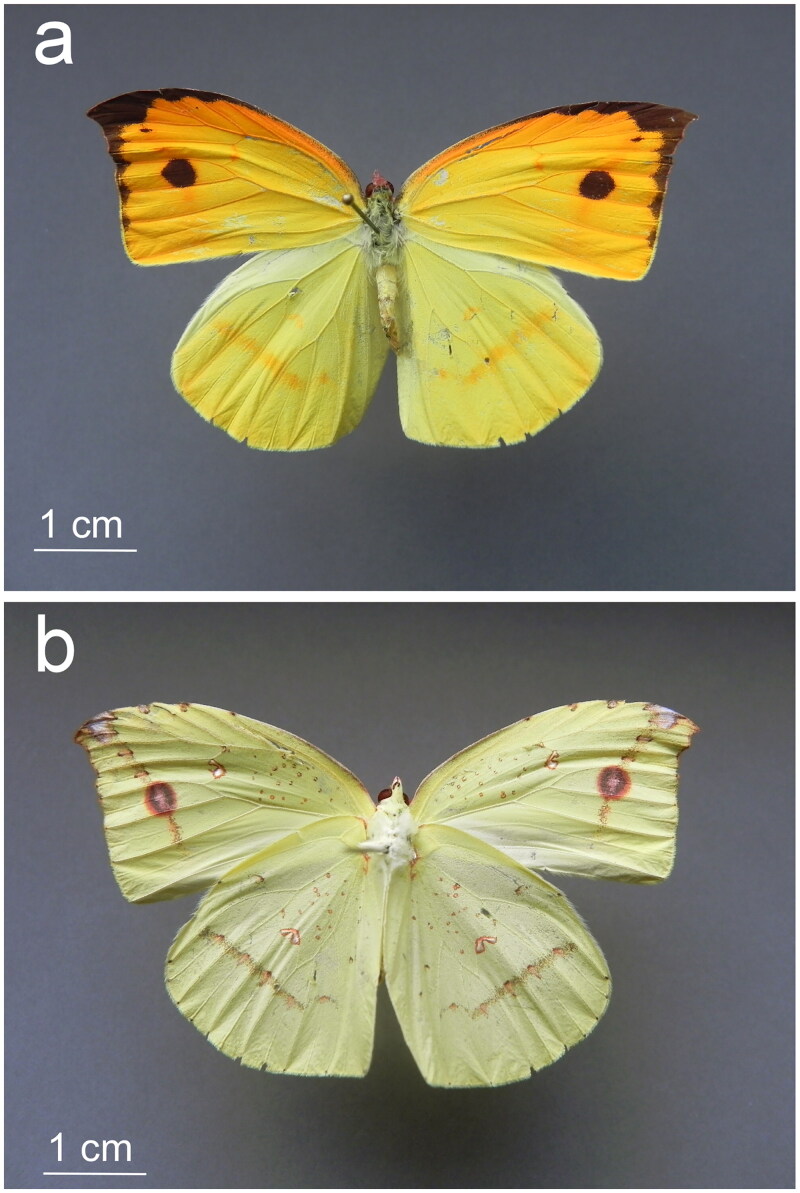
Photographs of the (a) dorsal and (b) ventral aspects of the *D. nina* specimen sampled for DNA in this study (photographed by Jeffrey M. Marcus). A neutral 18% grey card was used for the image backdrop. A 1 cm scale bar is included for each image.

A specimen of *D. nina* (lab code DN2020.1) was collected in Guilin, Guangxi Zhuan Autonomous Region, China (GPS 25.2819 N, 110.2863 E) in July 2020. All animal sample collection protocols complied with the current laws of China. The specimen (Figure 1) was distinguished from congeners and identified using the original species description with accompanying key to the genus (Mell 1913) on the basis of morphological characteristics including: pointed apex of the forewing, dorsal forewings suffused with orange-red pigment, black costal edge dusted with yellowish scales basally, ventral forewings have a brown-red ‘seam line’ running from the forewing apex to vein M3, and a bright yellow dorsal hindwing with an orange-reddish tinged ‘seam line’ bending at M3. The seam lines correspond to the distal bands of the central symmetry system of the nymphalid ground plan (Nijhout 1991). The specimen was deposited in the Wallis Roughley Museum of Entomology, University of Manitoba (http://www.wallisroughley.ca/, Jason Gibbs, Jason.Gibbs@umanitoba.ca) as voucher number WRME0507742.

### 2.2. DNA sequencing and genome assembly

A leg was removed from the specimen and total genomic DNA was prepared using a DNeasy Blood and Tissue kit (Qiagen, Düsseldorf, Germany) following the standard animal tissue extraction protocol with the following modifications as previously described (McCullagh and Marcus 2015): First, tissue was ground up in 180 μL of tissue lysis buffer ATL (Qiagen, Hilden, Germany) using a mortar and pestle; next, 20 μL of protein kinase K (Qiagen, Hilden, Germany, 600 mU/mL) was added to the mixture and then incubated in a 55 °C water bath for 1 h. The remainder of the purification steps were conducted exactly as described by the Qiagen protocol. Upon protocol completion, extracted and resuspended DNA was evaluated for yield and quality on a NanoDrop 2000 spectrophotometer (1.9 ng DNA/μL; Thermo Scientific, Wilmington, DE) and a Qubit 2.0 fluorometer (1.6142 ng DNA/μL; Life Technologies, Carlsbad, CA). DNA was stored in Eppendorf tubes (Eppendorf, Hamburg, Germany) at −20 °C until required (Peters and Marcus 2017).

The DNA sample was sheared by sonication with an S220 Focused-Ultrasonicator (Covaris, Woburn, MA). The shotgun sequencing library was prepared using NEBNext Ultra II DNA Library Prep Kit for Illumina (New England Biolabs, Ipswich, MA) and later sequenced by Illumina NovaSeq6000 equipped with an S4 PE150 flow cell and paired end reagent kit (San Diego, CA) (Marcus 2018)

The mitogenome assembly was created using Geneious Prime 2023.1 software (Biomatters, Auckland, New Zealand) (Kearse et al. 2012) which assembled the sequence library against a *Dercas lycorias* mitogenome (GenBank OR263671) (Wei et al. 2022) reference sequence without filtering.

### 2.3. Annotation and analysis

Mitochondrial genes were initially identified within Geneious Prime by aligning the *D. nina* mitogenome assembly to the annotated *D. lycorias* reference mitogenome and transferring homologous annotations to the newly assembled sequence. All gene positions were verified by comparisons between the newly assembled and reference mitogenomes using the ‘Align Two Sequences blastn’ option within GenBank BLAST+ 2.15.0 (Camacho et al. 2009). Additionally, the structure and location of all tRNAs were verified using ARWEN v.1.2 (Laslett and Canbäck 2008). The structure of the 16S rRNA was modeled using RNAfold as implemented in the ViennaRNA Package 2.0 (Lorenz et al. 2011). Geneious Prime was used for manual adjustments of gene annotations for start/stop codons and Proksee (Grant et al. 2023) was used to create the circular mitogenome map.

Phylogenetic analysis included the complete mitogenome of *D. nina*, along with the sole previously published *Dercas* mitogenome available from GenBank (from *D. lycorias*, OR263671; Wei et al. 2022), and a representative mitogenome from one species from each of the 14 other pierid genera with a previously reported complete mitogenome. To avoid making assumptions about the sister taxon to the Pieridae, we also included mitogenomes from 19 species representing the major clades of the six other butterfly families (Hedylidae, Hesperiidae, Lycaenidae, Nymphalidae, Papilionidae, and Riodinidae) (Table 1) (McCullagh et al. 2020). Three of these species in family Papilionidae were used as the outgroup to root the phylogenetic tree. Mitogenome sequences were aligned in CLUSTAL Omega (Sievers et al. 2011) and analyzed using Bayesian inference with the GTR + I + G model (model selected using jModeltest 2.1.1; Darriba et al. 2012) in MrBayes version 3.2.7a (Ronquist and Huelsenbeck 2003; Ronquist et al. 2012). Bayesian’s phylogenetic analysis included two runs consisting of three hot chains and one cold chain for 10 million iterations with sampling every 1000 generations.

**Table 1.** List of 35 butterfly species, GenBank accession numbers, specimen origin, family, and reference for sequences used in reconstruction of phylogenetic trees (Figure 3).

Species name	GenBank accession number	Specimen origin	Family	Reference
*Acraea zetes*	KT371361	UK	Nymphalidae	Timmermans, Lees, et al. (2016)
*Ancema ctesia*	ON710999	China	Lycaenidae	Liu C (unpublished)
*Anthocharis mandschurica*	MT499329	China	Pieridae: Pierinae	Zhou et al. (2020)
*Apodemia mormo*	KJ647171	N America	Riodinidae	Kim and Kim (2016)
*Aporia hastata*	OP373108	China	Pieridae: Pierinae	Jia et al. (2023)
*Apostictopterus fuliginosus*	MH985707	China	Hesperiidae	Han et al. (2018)
*Appias lalage*	MF576060	China	Pieridae: Pierinae	Zhang et al. (2019)
*Baltia butleri*	MH380204	China	Pieridae: Pierinae	Nie et al. (2018)
*Carterocephalus silvicola*	KJ629163	Korea	Hesperiidae	Kim et al. (2014)
*Cepora nadina*	OP779722	China	Pieridae: Pierinae	Wei et al. (2022)
*Coenonympha tullia*	KM592972	China	Nymphalidae	Timmermans, Viberg, et al. (2016)
*Colias erate*	KP715146	China	Pieridae: Coliadinae	Wu et al. (2016)
*Curetis acuta*	MZ196213	China	Lycaenidae	Weng Q (unpublished)
*Delias pasithoe*	MK252291	China	Pieridae: Pierinae	Wang J (unpublished)
*Dercas lycorias*	OR263671	China	Pieridae: Coliadinae	Wei et al. (2022)
*Dercas nina*	OR797085	China	Pieridae: Coliadinae	This study
*Dichorragia nesimachus*	KF590541	Taiwan	Nymphalidae	Wu et al. (2014)
*Dodona eugenes*	MT890732	China	Riodinidae	Wei et al. (2021)
*Gonepteryx amintha*	OP526832	China	Pieridae: Coliadinae	Zhao Z (unpublished)
*Hamadryas epinome*	KM378244	Peru	Nymphalidae	Cally et al. (2016)
*Ixias pyrene*	OP779726	China	Pieridae: Pierinae	Wei et al. (2022)
*Junonia lemonias*	KP941756	China	Nymphalidae	McCullagh and Marcus (2015)
*Limenitis sydyi*	KY593939	Taiwan	Nymphalidae	Chen et al. (2018)
*Macrosoma conifera*	MT852025	Costa Rica	Hedylidae	McCullagh et al. (2020)
*Pachliopta aristolochiae*	KU950357	China	Papilionidae	Li X, Xin T and Xia B (unpublished)
*Papilio demoleus*	KR024009	China	Papilionidae	Niu et al. (2016)
*Pareronia anais*	OP779723	China	Pieridae: Pierinae	Wei et al. (2022)
*Parnassius apollo*	KF746065	China	Papilionidae	Wang et al. (2015)
*Pieris napi*	MT576638	China	Pieridae: Pierinae	Yu et al. (2020)
*Polyommatus amorata*	ON411620	China	Lycaenidae	Chen WT (unpublished)
*Polyura arja*	KF590540	China	Nymphalidae	Wu et al. (2014)
*Pontia daplidice*	MH380207	China	Pieridae: Pierinae	Nie et al. (2018)
*Prioneris thestylis*	OP779724	China	Pieridae: Pierinae	Wei et al. (2022)
*Talbotia naganum*	MH380205	China	Pieridae: Pierinae	Nie et al. (2018)
*Zemeros flegyas*	MK521434	China	Riodinidae	Shi et al. (2020)

Convergence was determined using an effective sample size (ESS) estimation (which must exceed 625) as implemented in Convenience (Fabreti and Höhna 2022), and the first 2.5 million generations were discarded as burn-in. The resulting analysis resulted in an average deviation of split frequencies of 0.000348 and a mean estimated marginal likelihood of −193600.98.

## 3. Results

Two paired sequence libraries of 161,604,768 reads of 150 bp each (GenBank SRA SRR26257187) were created for *D. nina*. Two slightly different circular mitochondrial genome variants were assembled from 146,702 reads from these libraries which differ by only three SNPs, two 1-bp indels, and one 8-bp indel in a short region of the 16S rRNA. The phasing of this variation was possible because all of the observed polymorphisms occur within a span of no more than 101 bp and are detectable linked together within individual reads of the sequence library. RNAfold modeling of 16S structure shows that this variation occurs as length variation of a single helix and size-variation in the associated terminal loop within domain II of this rRNA.

The more common mitogenome variant 1 was 15,254 bp long and lacked nucleotides at all of the indel sites. The somewhat rarer mitogenome variant 2 was 15,264 bp and had additional nucleotides present in all three indel locations. The mitochondrial genome was reported to GenBank as accession OR797085, as the consensus of these two variants with the locations of the SNPs indicated by assigning degenerate nucleotide code symbols, while the positions of the indels are indicated by N’s in the overall consensus sequence. An alignment of the two variant 16S rRNA sequences with the consensus is provided in Supplementary Figure 1, while each of these 16S rRNA sequences is provided in FASTA format in Supplementary Figure 2. Depictions of the predicted structures of each of the variants are included in Supplementary Figure 3. The assembled consensus sequence was composed of 146,702 reads with nucleotide composition: 40.2% A, 10.6% C, 7.3% G, and 41.9% T. Consensus assembly mitogenome sequence coverage was 100% with a mean depth of coverage of 1471.3-fold (minimum 698-fold, maximum 2215-fold, Supplementary Figure 4).

**Figure 2.**
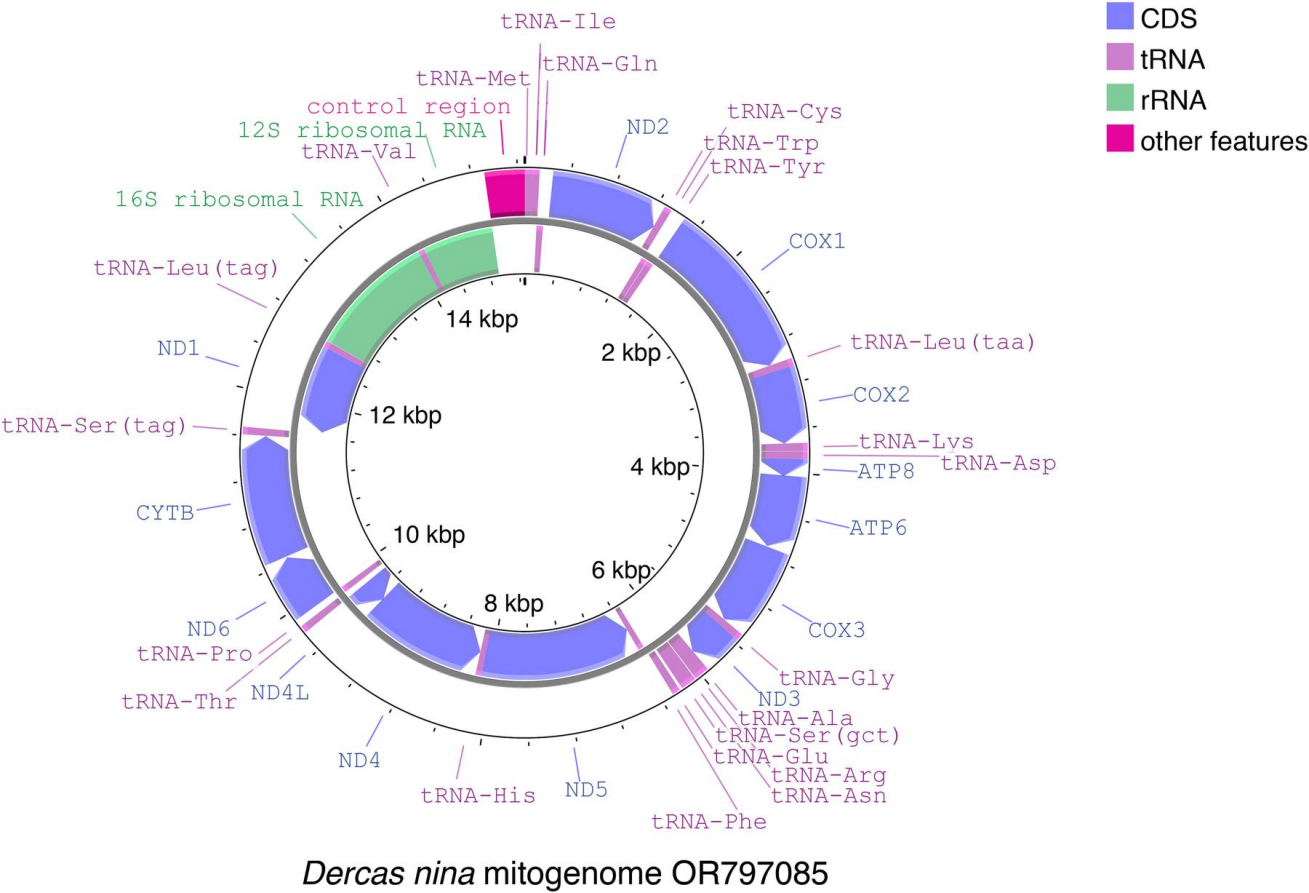
Circular mitochondrial genome feature map of *D. nina* created using Proksee software (Grant et al. 2023). Protein-coding genes are labeling in blue, tRNAs are labeled in purple, rRNAs are labeled in green, and the predicted control region is labeled in pink.

The gene composition and order of the *D. nina* mitogenome (Figure 2) is identical to that of most butterfly mitogenomes. The *D. nina* protein coding gene start codons include: ATG (*ATP6*, *COX2*, *COX3*, *CYTB*, *ND1*, *ND4*, *ND4L*), ATT (*ATP8*, *ND2*, *ND3*, *ND5*, *ND6*), and CGA, an atypical *COX1* start codon also found in *COX1* in many other insects (Liao et al. 2010). Six protein-coding genes (*COX1*, *COX2*, *ND2*, *ND3*, *ND4*, *ND5*) with predicted single-nucleotide (T) stop codons, and two protein-coding genes (*ATP6*, *ATP8*) with predicted two-nucleotide (TA) stop codons may be completed by post-transcriptional addition of 3′ A residues from the Poly-A tail. The predicted control region and mitochondrial rRNAs are typical for Lepidoptera, while the tRNAs have typical cloverleaf secondary structures except for *trn-Ser(act)* where the dihydrouridine arm has been replaced with a loop. A putative 7 bp lepidopteran mitochondrial transcription terminator (mtTERM) binding site (ATACTAA) was detected between *tRNA-Ser(tga)* and *ND1*.

**Figure 3.**
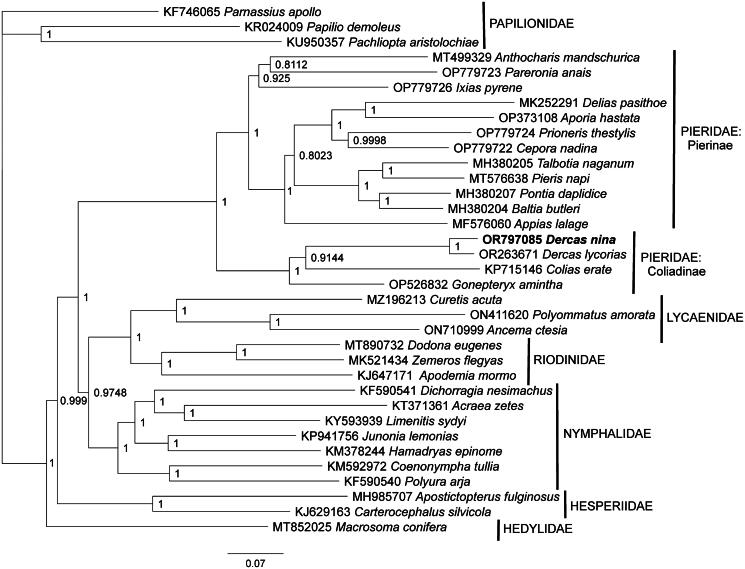
Bayesian’s inference phylogeny (GTR + I + G model, average deviation of split frequencies = 0.000348, mean estimated marginal likelihood = −193600.98) of the *Dercas nina* mitogenome, 15 additional mitogenomes from family Pieridae, and 19 species from six other butterfly families (Table 1). Three species in family Papilionidae were used as the phylogenetic outgroup to root the tree. The tree was produced by 10 million iterations in MrBayes with sampling every 1000 generations. At each node, the Bayesian posterior probability values determined by MrBayes are given. The scale bar depicts an average number of 0.7 substitutions per site per unit length.

Phylogenetic analysis (Figure 3) placed the *D. nina* mitogenome as sister to that of *D. lycorias. Dercas* mitogenomes were found as sister to *Colias* mitogenomes, with a *Gonepteryx* mitogenome as the outgroup within pierid subfamily Coliadinae. The availability of complete mitogenomes from both *D. nina* and *D. lycorias* provides an opportunity to make a variety of sequence comparisons between these congeners. The complete mitogenomes (OR797085 and OR263671) show an overall 96.17% sequence identity. Comparing just the complete *COX1* coding sequences from these accessions, which is often used for phylogenetic analysis, shows a 97.00% sequence identity. Focusing on just the DNA barcode region of *COX1*, which is often used for specimen identification, there is between 96.80% and 97.54% sequence identity between the barcode region of *D. nina* (OR797085) and five barcode sequences from *D. lycorias* (GenBank: OR263671, OR965399, ON436245; BOLD: VNMB2893-24, VNMB2894-24).

## 4. Discussion and conclusions

The *D. nina* mitogenome contains many structural features that make it similar to the mitogenomes of many other Lepidoptera, including a conserved gene arrangement (Park et al. 2016), a *trn-Ser(AGN)* where the dihydrouridine arm has been replaced with a loop (McCullagh and Marcus 2015), and the presence of a canonical 7 bp lepidopteran mtTERM binding site (ATACTAA) (Cameron and Whiting 2008; Gong et al. 2012) between *tRNA-Ser(TCR)* and *ND1*. The two 16S rRNA variant genotypes detected in the mitogenome assembly for *D. nina* differ only slightly and do not disrupt either the fine-scale or the overall structure of the resulting rRNAs, so we anticipate that both variants encode functional gene products.

The similarity in the levels of sequence identity between *D. nina* and *D. lycorias* complete mitogenomes, complete *COX1* coding sequences, and *COX1* barcode regions is consistent with prior observations in other taxa (Peters and Marcus 2017). This suggests that the amount of sequence identity in the *COX1* DNA barcode region between two species might be useful as a predictor of the degree of sequence identity between their entire mitogenomes.

Phylogenetic analysis found genus *Dercas* mitogenomes as monophyletic, as might be predicted based on taxonomy. Contrary to the morphology-based predictions of Schulze and Fiedler (1997), *Dercas* mitogenomes were not found to be sister to the mitogenome from *Gonepteryx*, but rather *Dercas* mitogenomes were sister to *Colias* mitogenomes, with *Gonepteryx* as an outgroup, which is more consistent with some previous molecular phylogenetic analyses based on other molecular markers (Ding and Zhang 2017; Wei et al. 2022). Whether this finding should be interpreted as an experimental artifact attributable to limited representation of mitogenomes from genera in the subfamily Coliadinae in the phylogenetic analysis, as evidence falsifying the *Dercas-Gonepteryx* sister clade hypothesis, or whether it reflects mitochondrial-nuclear phylogenetic discordance within the Coliadinae should be determined through additional investigations.

## Supplementary Material

Dercas nina Mitogenome Supplemental Figures October2024.docx

## Data Availability

The genome sequence data that support the findings of this study are openly available in GenBank of NCBI at https://www.ncbi.nlm.nih.gov/ under the accession no. OR797085. The associated BioProject, SRA, and Bio-Sample numbers are PRJNA1023248, SRR26257187, and SAMN37648387, respectively.
