# Supplementary material for: Phylogenetic analysis of the complete mitochondrial genome of the orange-winged sulphur butterfly Dercas nina Mell 1913 (Insecta: Lepidoptera: Pieridae: Coliadinae)
Source: Mitochondrial DNA B Resour. 2024 Nov 13;9(11):1510–7. doi: 10.1080/23802359.2024.2427109 (PMC11562023; doi:10.1080/23802359.2024.2427109)

Supplementary Figure 1. Clustal Omega alignment of mitochondrial 16S rRNA sequence variants in *Dercas nina.* The Variant 1 mitogenome is 15,254 bp long, while the Variant 2 mitogenome is 15,264 bp long. The *D. nina* consensus mitogenome sequence as reported in GenBank accession OR797085 is 15,264 bp, with degenerate nucleotides in the SNP positions and N’s in the location of each indel. These positions are further highlighted in yellow here, with red text used to identify the unique nucleotides in each of the variants.

CLUSTAL O(1.2.4) multiple sequence alignment

16S_rRNA_variant1 TAATTTTAATAATATTCTTAAAAATTAAATTATTCAAAATAATTAATCCTTTCGTACTAA 60

16S_rRNA_OR797085 TAATTTTAATAATATTCTTAAAAATTAAATTATTCAAAATAATTAATCCTTTCGTACTAA 60

16S_rRNA_variant2 TAATTTTAATAATATTCTTAAAAATTAAATTATTCAAAATAATTAATCCTTTCGTACTAA 60

************************************************************

16S_rRNA_variant1 ATTATTTTATTTATATAAAGATAGAAACCAACCTGGCTTACACCGGTTTGAACTCAGATC 120

16S_rRNA_OR797085 ATTATTTTATTTATATAAAGATAGAAACCAACCTGGCTTACACCGGTTTGAACTCAGATC 120

16S_rRNA_variant2 ATTATTTTATTTATATAAAGATAGAAACCAACCTGGCTTACACCGGTTTGAACTCAGATC 120

************************************************************

16S_rRNA_variant1 ATGTAAGATTTTAATGATCGAACAGATCAAAAATATTAAACTTTTGCATTTAAATTTTAT 180

16S_rRNA_OR797085 ATGTAAGATTTTAATGATCGAACAGATCAAAAATATTAAACTTTTGCATTTAAATTTTAT 180

16S_rRNA_variant2 ATGTAAGATTTTAATGATCGAACAGATCAAAAATATTAAACTTTTGCATTTAAATTTTAT 180

************************************************************

16S_rRNA_variant1 CTTAATCCAACATCGAGGTCGCAAACTTTCTTTTTTATTTGAACTAAAAAAAAAAATTAC 240

16S_rRNA_OR797085 CTTAATCCAACATCGAGGTCGCAAACTTTCTTTTTTATTTGAACTAAAAAAAAAAATTAC 240

16S_rRNA_variant2 CTTAATCCAACATCGAGGTCGCAAACTTTCTTTTTTATTTGAACTAAAAAAAAAAATTAC 240

************************************************************

16S_rRNA_variant1 GCTGTTATCCCTAAGGTAATTTATTCTTATAATCATAAATTATGGATCAAATATTCATAT 300

16S_rRNA_OR797085 GCTGTTATCCCTAAGGTAATTTATTCTTATAATCATAAATTATGGATCAAATATTCATAT 300

16S_rRNA_variant2 GCTGTTATCCCTAAGGTAATTTATTCTTATAATCATAAATTATGGATCAAATATTCATAT 300

************************************************************

16S_rRNA_variant1 ATTAATGTTAAATTTAAAAAAAAGTTATTTTTATTTTTTTATCACCCCAACAAAATAATT 360

16S_rRNA_OR797085 ATTAATGTTAAATTTAAAAAAAAGTTATTTTTATTTTTTTATCACCCCAACAAAATAATT 360

16S_rRNA_variant2 ATTAATGTTAAATTTAAAAAAAAGTTATTTTTATTTTTTTATCACCCCAACAAAATAATT 360

************************************************************

16S_rRNA_variant1 ATTTAATTATAATGATTAATTTTTATAAATAAATTATAATTAAATAATTATAAAACTCTA 420

16S_rRNA_OR797085 ATTTAATTATAATGATTAATTTTTATAAATAAATTATAATTAAATAATTATAAAACTCTA 420

16S_rRNA_variant2 ATTTAATTATAATGATTAATTTTTATAAATAAATTATAATTAAATAATTATAAAACTCTA 420

************************************************************

16S_rRNA_variant1 TAGGGTCTTCTCGTCTTTTATAATTATTTTAGCTTTTTAACTAAAAAATTAAATTCTAAT 480

16S_rRNA_OR797085 TAGGGTCTTCTCGTCTTTTATAATTATTTTAGCTTTTTAACTAAAAAATTAAATTCTAAT 480

16S_rRNA_variant2 TAGGGTCTTCTCGTCTTTTATAATTATTTTAGCTTTTTAACTAAAAAATTAAATTCTAAT 480

************************************************************

16S_rRNA_variant1 TTTAAAATAGAAACAGTTTATATTTCATTAAATCATTCATACAAGTCTCCAATTAAAAGA 540

16S_rRNA_OR797085 TTTAAAATAGAAACAGTTTATATTTCATTAAATCATTCATACAAGTCTCCAATTAAAAGA 540

16S_rRNA_variant2 TTTAAAATAGAAACAGTTTATATTTCATTAAATCATTCATACAAGTCTCCAATTAAAAGA 540

************************************************************

16S_rRNA_variant1 CTAATGATTATGCTACCTTTGTACAGTCAATATACTGCAGCCCTTTAAATTAATTCAGTG 600

16S_rRNA_OR797085 CTAATGATTATGCTACCTTTGTACAGTCAATATACTGCAGCCCTTTAAATTAATTCAGTG 600

16S_rRNA_variant2 CTAATGATTATGCTACCTTTGTACAGTCAATATACTGCAGCCCTTTAAATTAATTCAGTG 600

************************************************************

16S_rRNA_variant1 GGCAGATTAGACTTTATATTATTTTCAAAAAGACATGTTTTTGATAAACAGGTGAATATA 660

16S_rRNA_OR797085 GGCAGATTAGACTTTATATTATTTTCAAAAAGACATGTTTTTGATAAACAGGTGAATATA 660

16S_rRNA_variant2 GGCAGATTAGACTTTATATTATTTTCAAAAAGACATGTTTTTGATAAACAGGTGAATATA 660

************************************************************

16S_rRNA_variant1 TAATTTTGCCGAATTCTTTTATTTTAATTATAAAAATTTTTATATTATAATTAAAAATAT 720

16S_rRNA_OR797085 TAATTTTGCCGAATTCTTTTATTTTAATTATAAAAATTTTTATATTATAATTAAAAATAT 720

16S_rRNA_variant2 TAATTTTGCCGAATTCTTTTATTTTAATTATAAAAATTTTTATATTATAATTAAAAATAT 720

************************************************************

16S_rRNA_variant1 ACTAATTTTATCATTATTTCTAATTTTATATTATTATATATTATTTTTTTATAAAAAATT 780

16S_rRNA_OR797085 ACTAATTTTATCATTATTTCTAATTTTATATTATTATATATTATTTTTTTATAAAAAATT 780

16S_rRNA_variant2 ACTAATTTTATCATTATTTCTAATTTTATATTATTATATATTATTTTTTTATAAAAAATT 780

************************************************************

16S_rRNA_variant1 AAATTAATTTATATAAAATTTTTAATTAAATAAAATTATTTATAAAAAATTATAATTTAT 840

16S_rRNA_OR797085 AAATTAATTTATATAAAATTTTTAATTAAATAAAATTATTTATAAAAAATTATAATTTAT 840

16S_rRNA_variant2 AAATTAATTTATATAAAATTTTTAATTAAATAAAATTATTTATAAAAAATTATAATTTAT 840

************************************************************

16S_rRNA_variant1 AAAAAATATAATTTTTAAAAATTTTATTTATATATATAAAAAAAATAATAAATATAT-AA 900

16S_rRNA_OR797085 AAAAAATATAATTTTTAAAAATTTTATTTATATATATAAAAAAAATAAWWAATATATNAA 900

16S_rRNA_variant2 AAAAAATATAATTTTTAAAAATTTTATTTATATATATAAAAAAAATAAATAATATATAAA 900

************************************************ ******* **

16S_rRNA_variant1 AAATAT-TATTTAAATATATAAATATATTATAAAATTCTAATTTAAAGCTTATCCTTTAA 958

16S_rRNA_OR797085 AAATATNTATTTAAATATATAAATATATTATAAAATTYTAATTTAAAGCTTATCCTTTAA 960

16S_rRNA_variant2 AAATATTTATTTAAATATATAAATATATTATAAAATTTTAATTTAAAGCTTATCCTTTAA 960

****** ****************************** **********************

16S_rRNA_variant1 AATATTTAATTAATTTTATTT--------TTTTTTAATAAATAAAAAAAAAATTAAAATT 1010

16S_rRNA_OR797085 AATATTTAATTAATTTTATTTNNNNNNNNTTTTTTAATAAATAAAAAAAAAATTAAAATT 1020

16S_rRNA_variant2 AATATTTAATTAATTTTATTTATTTTATTTTTTTTAATAAATAAAAAAAAAATTAAAATT 1020

********************* *******************************

16S_rRNA_variant1 AATTAAAAATTTAATTTTTTTCTAAAAAAACTAGATATATTTAAAAACGATTAACATTTC 1070

16S_rRNA_OR797085 AATTAAAAATTTAATTTTTTTCTAAAAAAACTAGATATATTTAAAAACGATTAACATTTC 1080

16S_rRNA_variant2 AATTAAAAATTTAATTTTTTTCTAAAAAAACTAGATATATTTAAAAACGATTAACATTTC 1080

************************************************************

16S_rRNA_variant1 ATTTCAAATTAATTATTAAAAATAATTATACAACATTAAATTTACTAATTAATTATCTCT 1130

16S_rRNA_OR797085 ATTTCAAATTAATTATTAAAAATAATTATACAACATTAAATTTACTAATTAATTATCTCT 1140

16S_rRNA_variant2 ATTTCAAATTAATTATTAAAAATAATTATACAACATTAAATTTACTAATTAATTATCTCT 1140

************************************************************

16S_rRNA_variant1 TTTAAATTCGAGATATTTTTTTTTAAAAATAATTATTAAATAAACTCTGATACACAAGAT 1190

16S_rRNA_OR797085 TTTAAATTCGAGATATTTTTTTTTAAAAATAATTATTAAATAAACTCTGATACACAAGAT 1200

16S_rRNA_variant2 TTTAAATTCGAGATATTTTTTTTTAAAAATAATTATTAAATAAACTCTGATACACAAGAT 1200

************************************************************

16S_rRNA_variant1 ACAATAAATAAAATTTACTTATTATTTAATATATTTTTAATATATATATTTCAAAATTCT 1250

16S_rRNA_OR797085 ACAATAAATAAAATTTACTTATTATTTAATATATTTTTAATATATATATTTCAAAATTCT 1260

16S_rRNA_variant2 ACAATAAATAAAATTTACTTATTATTTAATATATTTTTAATATATATATTTCAAAATTCT 1260

************************************************************

16S_rRNA_variant1 TTCACAATACTATTATACTATTAATAAAAAAAAAATTTTTTCTAAAATATACTTTAACCC 1310

16S_rRNA_OR797085 TTCACAATACTATTATACTATTAATAAAAAAAAAATTTTTTCTAAAATATACTTTAACCC 1320

16S_rRNA_variant2 TTCACAATACTATTATACTATTAATAAAAAAAAAATTTTTTCTAAAATATACTTTAACCC 1320

************************************************************

16S_rRNA_variant1 CCATTAAAATTATTTTATTAAAATTATTTTTATTAAATATTTATCTTATTAATTTATTTT 1370

16S_rRNA_OR797085 CCATTAAAATTATTTTATTAAAATTATTTTTATTAAATATTTATCTTATTAATTTATTTT 1380

16S_rRNA_variant2 CCATTAAAATTATTTTATTAAAATTATTTTTATTAAATATTTATCTTATTAATTTATTTT 1380

************************************************************

16S_rRNA_variant1 CA 1372

16S_rRNA_OR797085 CA 1382

16S_rRNA_variant2 CA 1382

**

Supplementary Figure 2. Sequences of mitochondrial 16S rRNA sequence variants and the consensus sequences of the 16S rRNA in *Dercas nina* in the same orientation as reported in GenBank accession OR797085, which is the reverse complement to the direction in which this gene is transcribed in the mitochondrion.

>16S_rRNA_OR797085

TAATTTTAATAATATTCTTAAAAATTAAATTATTCAAAATAATTAATCCTTTCGTACTAAATTATTTTATTTATATAAAGATAGAAACCAACCTGGCTTACACCGGTTTGAACTCAGATCATGTAAGATTTTAATGATCGAACAGATCAAAAATATTAAACTTTTGCATTTAAATTTTATCTTAATCCAACATCGAGGTCGCAAACTTTCTTTTTTATTTGAACTAAAAAAAAAAATTACGCTGTTATCCCTAAGGTAATTTATTCTTATAATCATAAATTATGGATCAAATATTCATATATTAATGTTAAATTTAAAAAAAAGTTATTTTTATTTTTTTATCACCCCAACAAAATAATTATTTAATTATAATGATTAATTTTTATAAATAAATTATAATTAAATAATTATAAAACTCTATAGGGTCTTCTCGTCTTTTATAATTATTTTAGCTTTTTAACTAAAAAATTAAATTCTAATTTTAAAATAGAAACAGTTTATATTTCATTAAATCATTCATACAAGTCTCCAATTAAAAGACTAATGATTATGCTACCTTTGTACAGTCAATATACTGCAGCCCTTTAAATTAATTCAGTGGGCAGATTAGACTTTATATTATTTTCAAAAAGACATGTTTTTGATAAACAGGTGAATATATAATTTTGCCGAATTCTTTTATTTTAATTATAAAAATTTTTATATTATAATTAAAAATATACTAATTTTATCATTATTTCTAATTTTATATTATTATATATTATTTTTTTATAAAAAATTAAATTAATTTATATAAAATTTTTAATTAAATAAAATTATTTATAAAAAATTATAATTTATAAAAAATATAATTTTTAAAAATTTTATTTATATATATAAAAAAAATAAWWAATATATNAAAAATATNTATTTAAATATATAAATATATTATAAAATTYTAATTTAAAGCTTATCCTTTAAAATATTTAATTAATTTTATTTNNNNNNNNTTTTTTAATAAATAAAAAAAAAATTAAAATTAATTAAAAATTTAATTTTTTTCTAAAAAAACTAGATATATTTAAAAACGATTAACATTTCATTTCAAATTAATTATTAAAAATAATTATACAACATTAAATTTACTAATTAATTATCTCTTTTAAATTCGAGATATTTTTTTTTAAAAATAATTATTAAATAAACTCTGATACACAAGATACAATAAATAAAATTTACTTATTATTTAATATATTTTTAATATATATATTTCAAAATTCTTTCACAATACTATTATACTATTAATAAAAAAAAAATTTTTTCTAAAATATACTTTAACCCCCATTAAAATTATTTTATTAAAATTATTTTTATTAAATATTTATCTTATTAATTTATTTTCA

>16S_rRNA_variant1

TAATTTTAATAATATTCTTAAAAATTAAATTATTCAAAATAATTAATCCTTTCGTACTAAATTATTTTATTTATATAAAGATAGAAACCAACCTGGCTTACACCGGTTTGAACTCAGATCATGTAAGATTTTAATGATCGAACAGATCAAAAATATTAAACTTTTGCATTTAAATTTTATCTTAATCCAACATCGAGGTCGCAAACTTTCTTTTTTATTTGAACTAAAAAAAAAAATTACGCTGTTATCCCTAAGGTAATTTATTCTTATAATCATAAATTATGGATCAAATATTCATATATTAATGTTAAATTTAAAAAAAAGTTATTTTTATTTTTTTATCACCCCAACAAAATAATTATTTAATTATAATGATTAATTTTTATAAATAAATTATAATTAAATAATTATAAAACTCTATAGGGTCTTCTCGTCTTTTATAATTATTTTAGCTTTTTAACTAAAAAATTAAATTCTAATTTTAAAATAGAAACAGTTTATATTTCATTAAATCATTCATACAAGTCTCCAATTAAAAGACTAATGATTATGCTACCTTTGTACAGTCAATATACTGCAGCCCTTTAAATTAATTCAGTGGGCAGATTAGACTTTATATTATTTTCAAAAAGACATGTTTTTGATAAACAGGTGAATATATAATTTTGCCGAATTCTTTTATTTTAATTATAAAAATTTTTATATTATAATTAAAAATATACTAATTTTATCATTATTTCTAATTTTATATTATTATATATTATTTTTTTATAAAAAATTAAATTAATTTATATAAAATTTTTAATTAAATAAAATTATTTATAAAAAATTATAATTTATAAAAAATATAATTTTTAAAAATTTTATTTATATATATAAAAAAAATAATAAATATATAAAAATATTATTTAAATATATAAATATATTATAAAATTCTAATTTAAAGCTTATCCTTTAAAATATTTAATTAATTTTATTTTTTTTTAATAAATAAAAAAAAAATTAAAATTAATTAAAAATTTAATTTTTTTCTAAAAAAACTAGATATATTTAAAAACGATTAACATTTCATTTCAAATTAATTATTAAAAATAATTATACAACATTAAATTTACTAATTAATTATCTCTTTTAAATTCGAGATATTTTTTTTTAAAAATAATTATTAAATAAACTCTGATACACAAGATACAATAAATAAAATTTACTTATTATTTAATATATTTTTAATATATATATTTCAAAATTCTTTCACAATACTATTATACTATTAATAAAAAAAAAATTTTTTCTAAAATATACTTTAACCCCCATTAAAATTATTTTATTAAAATTATTTTTATTAAATATTTATCTTATTAATTTATTTTCA

>16S_rRNA_variant2

TAATTTTAATAATATTCTTAAAAATTAAATTATTCAAAATAATTAATCCTTTCGTACTAAATTATTTTATTTATATAAAGATAGAAACCAACCTGGCTTACACCGGTTTGAACTCAGATCATGTAAGATTTTAATGATCGAACAGATCAAAAATATTAAACTTTTGCATTTAAATTTTATCTTAATCCAACATCGAGGTCGCAAACTTTCTTTTTTATTTGAACTAAAAAAAAAAATTACGCTGTTATCCCTAAGGTAATTTATTCTTATAATCATAAATTATGGATCAAATATTCATATATTAATGTTAAATTTAAAAAAAAGTTATTTTTATTTTTTTATCACCCCAACAAAATAATTATTTAATTATAATGATTAATTTTTATAAATAAATTATAATTAAATAATTATAAAACTCTATAGGGTCTTCTCGTCTTTTATAATTATTTTAGCTTTTTAACTAAAAAATTAAATTCTAATTTTAAAATAGAAACAGTTTATATTTCATTAAATCATTCATACAAGTCTCCAATTAAAAGACTAATGATTATGCTACCTTTGTACAGTCAATATACTGCAGCCCTTTAAATTAATTCAGTGGGCAGATTAGACTTTATATTATTTTCAAAAAGACATGTTTTTGATAAACAGGTGAATATATAATTTTGCCGAATTCTTTTATTTTAATTATAAAAATTTTTATATTATAATTAAAAATATACTAATTTTATCATTATTTCTAATTTTATATTATTATATATTATTTTTTTATAAAAAATTAAATTAATTTATATAAAATTTTTAATTAAATAAAATTATTTATAAAAAATTATAATTTATAAAAAATATAATTTTTAAAAATTTTATTTATATATATAAAAAAAATAAATAATATATAAAAAATATTTATTTAAATATATAAATATATTATAAAATTTTAATTTAAAGCTTATCCTTTAAAATATTTAATTAATTTTATTTATTTTATTTTTTTTAATAAATAAAAAAAAAATTAAAATTAATTAAAAATTTAATTTTTTTCTAAAAAAACTAGATATATTTAAAAACGATTAACATTTCATTTCAAATTAATTATTAAAAATAATTATACAACATTAAATTTACTAATTAATTATCTCTTTTAAATTCGAGATATTTTTTTTTAAAAATAATTATTAAATAAACTCTGATACACAAGATACAATAAATAAAATTTACTTATTATTTAATATATTTTTAATATATATATTTCAAAATTCTTTCACAATACTATTATACTATTAATAAAAAAAAAATTTTTTCTAAAATATACTTTAACCCCCATTAAAATTATTTTATTAAAATTATTTTTATTAAATATTTATCTTATTAATTTATTTTCA

Supplementary Figure 3. Structures of mitochondrial 16S rRNA sequence variant gene products in *Dercas nina* as predicted by RNAfold (Lorenz et al. 2011): (a) variant 1, (b) variant 2. Each of the domains conserved across nearly all mitochondrial rRNAs are indicated by Roman numberals (note that domain III does not occur in arthropods, so its location is labeled, but the Roman numeral appears in parentheses. The helix and terminal loop in conserved domain II that differs between the two variants is enlarged in blue text for ease of comparison between the two variants.


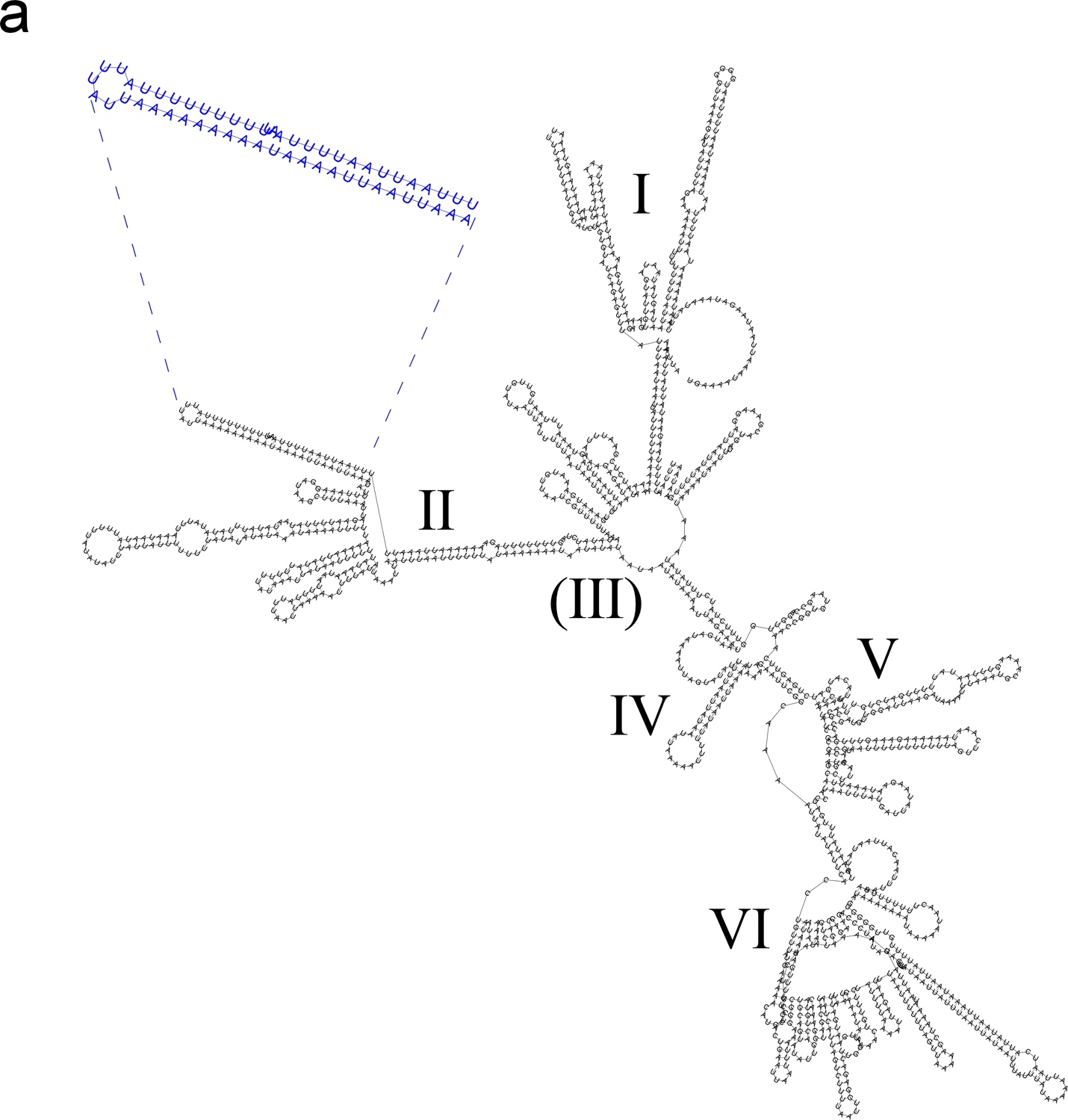


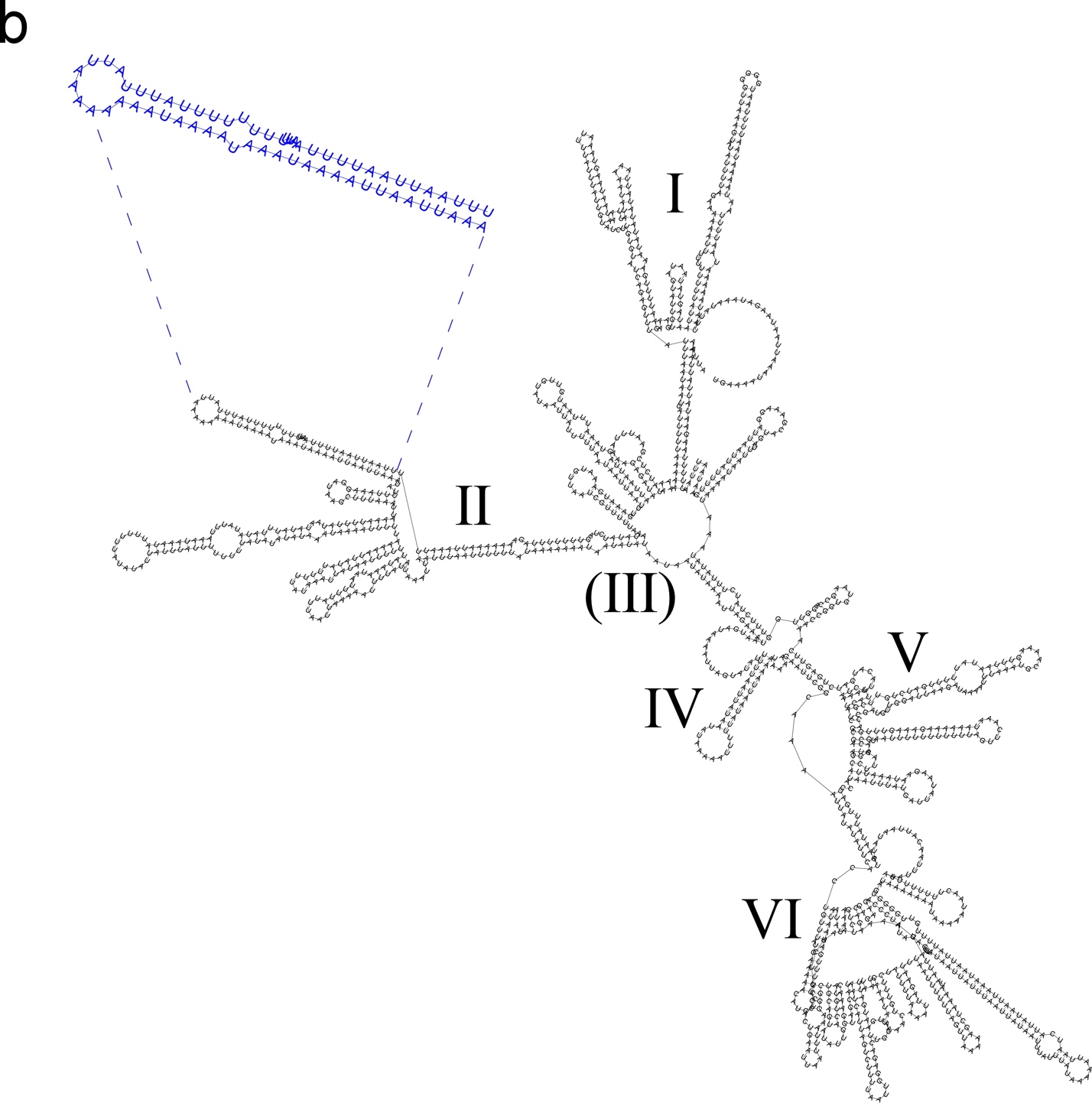


Supplementary Figure 4. Read coverage plot of the consensus mitogenome assembly for *Dercas nina.* Assembly mitogenome coverage was 100% with a mean depth of coverage of 1471.3-fold (minimum 698-fold, maximum 2215-fold).


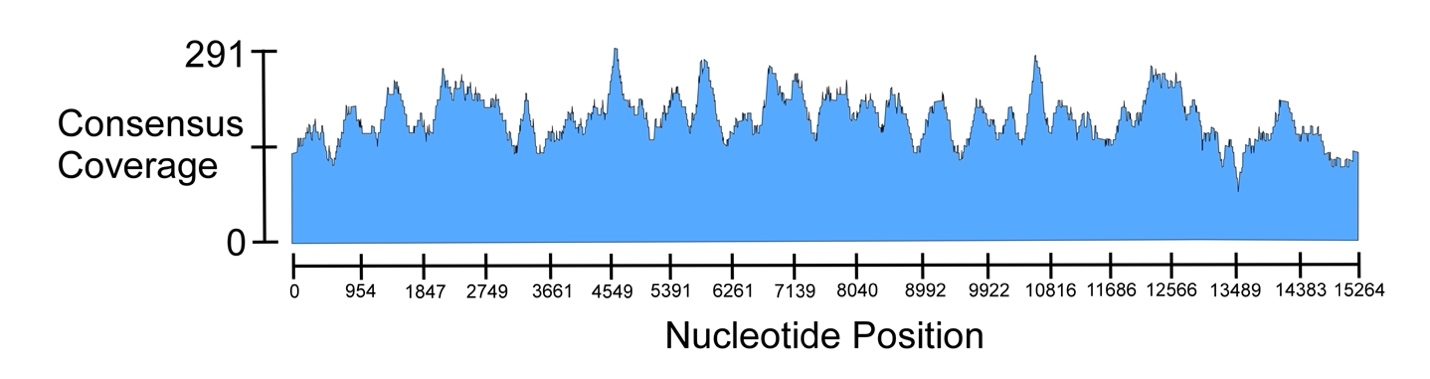

Supplement: Dercas nina Mitogenome Supplemental Figures October2024.docx [file TMDN_A_2427109_SM2406.docx]
